# A Crustin from Hydrothermal Vent Shrimp: Antimicrobial Activity and Mechanism

**DOI:** 10.3390/md19030176

**Published:** 2021-03-23

**Authors:** Yujian Wang, Jian Zhang, Yuanyuan Sun, Li Sun

**Affiliations:** 1 CAS and Shandong Province Key Laboratory of Experimental Marine Biology, Institute of Oceanology, Center for Ocean Mega-Science, Chinese Academy of Sciences, Qingdao 266071, China; wangyujian@qdio.ac.cn (Y.W.); zhangjian@ytu.edu.cn (J.Z.); sunyuanyuan@qdio.ac.cn (Y.S.); 2 Laboratory for Marine Biology and Biotechnology, Pilot National Laboratory for Marine Science and Technology (Qingdao), Qingdao 266237, China; 3 College of Earth and Planetary Sciences, University of Chinese Academy of Sciences, Beijing 100049, China; 4 School of Ocean, Yantai University, Yantai 264005, China

**Keywords:** crustin, antimicrobial peptides, shrimp, deep-sea hydrothermal vent

## Abstract

Crustin is a type of antimicrobial peptide and plays an important role in the innate immunity of arthropods. We report here the identification and characterization of a crustin (named Crus1) from the shrimp *Rimicaris* sp. inhabiting the deep-sea hydrothermal vent in Manus Basin (Papua New Guinea). Crus1 shares the highest identity (51.76%) with a Type I crustin of *Penaeus vannamei* and possesses a whey acidic protein (WAP) domain, which contains eight cysteine residues that form the conserved ‘four-disulfide core’ structure. Recombinant Crus1 (rCrus1) bound to peptidoglycan and lipoteichoic acid, and effectively killed Gram-positive bacteria in a manner that was dependent on pH, temperature, and disulfide linkage. rCrus1 induced membrane leakage and structure damage in the target bacteria, but had no effect on bacterial protoplasts. Serine substitution of each of the 8 Cys residues in the WAP domain did not affect the bacterial binding capacity but completely abolished the bactericidal activity of rCrus1. These results provide new insights into the characteristic and mechanism of the antimicrobial activity of deep sea crustins.

## 1. Introduction

Antimicrobial peptides (AMPs) are a class of evolutionarily conserved molecules that exist in almost all organisms as mediators of innate immunity. Invertebrates, which lack the adaptive immune system, rely particularly on AMPs and other innate immune factors to resist invading pathogens [1,2]. Functionally, AMPs can destroy invading microbial pathogens and directly kill bacteria, fungi, viruses, and parasites [3,4]. Unlike traditional antibiotics, which are well known to induce resistance in the target bacteria, AMPs are intrinsic components of organisms and target the inner and/or outer membranes of bacteria in a non-receptor-specific manner, with a rate of resistance several orders of magnitude lower than that of conventional antibiotics [5,6,7].

AMPs are highly diverse in structure and function, and usually have a low molecular mass (<10 kDa) [8]. AMPs possess biochemical features, such as amino acid composition, size, amphipathicity, and cationic charge, that allow them to have a high propensity for selective membrane-interaction [8]. Extensive studies indicate that AMP-mediated permeabilization/disruption of the microbial cytoplasmic membrane is the main mechanism of cell killing for most AMPs [9,10,11]. Some non-membrane permeable AMPs can inhibit or destroy the key processes of intracellular targets (DNA, RNA or protein), inactivate essential intracellular enzymes, or affect the formation of membrane compartments and cell wall synthesis [8,12,13,14].

Crustin is categorized as a type of AMP that plays a vital role in the immune defense of crustaceans. Crustin is generally a cationic peptide of 7–22 kDa and contains twelve conserved cysteine residues, eight of which comprise a typical whey acid protein (WAP) domain [15]. The WAP domain forms a four-disulfide bond core arrangement at the C-terminus and is potentially associated with multiple functions [16,17]. At present, a large number of crustins have been reported, which exhibit various antibacterial or protease inhibitory functions [18,19,20]. However, in many cases, the specific bactericidal mechanisms of the crustins remain to be investigated.

The deep sea is the largest ecosystem on earth, with many unique biological resources, including microorganisms and invertebrates [21,22,23]. Marine invertebrates, such as shrimp, have been considered as promising sources for the discovery of bioactive materials [24]. Shrimps of the family Alvinocarididae inhabit the deep waters in Atlantic, Pacific, and Indian Oceans, especially the hydrothermal vents and cold seeps, where they are often found to be the dominant fauna [25,26]. Recently, a novel anti-Gram-positive crustin, Re-crustin, was identified from the extremophile Pleocyemata shrimp, *Rimicaris exoculata*, collected from the hydrothermal vent site of the Mid-Atlantic Ridge (MAR) [27]. In a previous study, we identified several crustin-like genes from the transcriptome of the shrimp *Rimicaris* sp (Alvinocarididae family) from a hydrothermal vent in Desmos, manus basin [26]. In this study, we characterized one of these crustins (designated Crus1). We investigated the structural feature and antimicrobial effect of recombinant Crus1, and identified the key cysteine residues required for bactericidal activity. Our results add new knowledge to the antimicrobial mechanism of deep sea crustins.

## 2. Results

### 2.1. Sequence and Structure Characterization of Crus1

**Figure 1:**
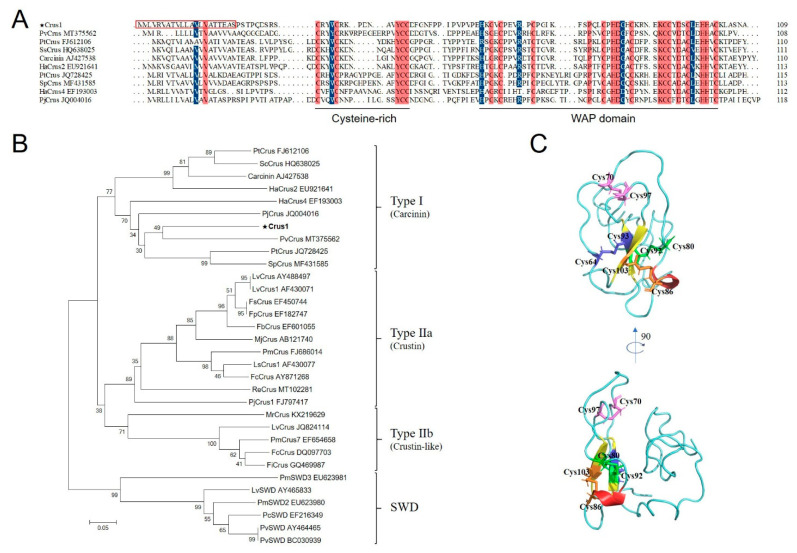
Sequence, phylogenetic, and structural analysis of Crus1. (**A**) Alignment of Crus1 with Type I crustins. Dots denote gaps introduced for maximum matching. The consensus residues are shaded red, the residues that are ≥75% identical among the aligned sequences are shaded blue. The signal peptide sequence of Crus1 is boxed with red lines. (**B**) Phylogenetic analysis of Crus1 homologues. The phylogenetic tree was constructed with MEGA 6.0 using the neighbor-joining method. Numbers beside the internal branches indicate bootstrap values based on 1000 replications. The GenBank accession numbers of the crustins used in (**A**,**B**) are indicated after the names of the crustins. (**C**) The predicted structure of Crus1 was built using I-TASSER. The disulfide bonds in the WAP domain are shown in blue (C64–C93), pink (C70–C97), green (C80–C92) and orange (C86–C103).

The deduced amino acid sequence of Crus1 contains 109 residues, with a calculated molecular weight of 12.05 kDa and a predicted pI of 7.82. Crus1 possesses a signal peptide in the N-terminus (residues 1 to 20) and a WAP domain in the C-terminus, in which a ‘four-disulfide core’ structure can be formed by C64–C93, C70–C97, C80–C92, and C86–C103. Protein BLAST showed that Crus1 shares the highest identity (51.76%) with PvCrus, a Type I crustin of *Penaeus vannamei* (GenBank accession No. MT375562). The sequence identity between Crus1 and Re-crustin, the Type II crustin identified in the shrimp from MAR [27], is 24.74%. The sequence alignment between Crus1 and representative Type I crustins indicated that the conserved WAP domain, in particular the 8 cysteines that form the four-disulfide core structure, was shared among the crustins (Figure 1A). In addition, four cysteines in the N-terminus (corresponding to C31, C35, C44, and C45 in Crus1) were also conserved among the Type I crustins (Figure 1A). Phylogenetic analysis showed that Crus1 was grouped into the clade of Type I crustin (Figure 1B). The predicted protein structure of Crus1 contains mostly random coils, with a very little amount of α-helix and β-pleated sheet (Figure 1C).

### 2.2. Antimicrobial Activity of rCrus1 and Its Dependence on Temperature, pH, and Disulfide Bonds

**Figure 2:**
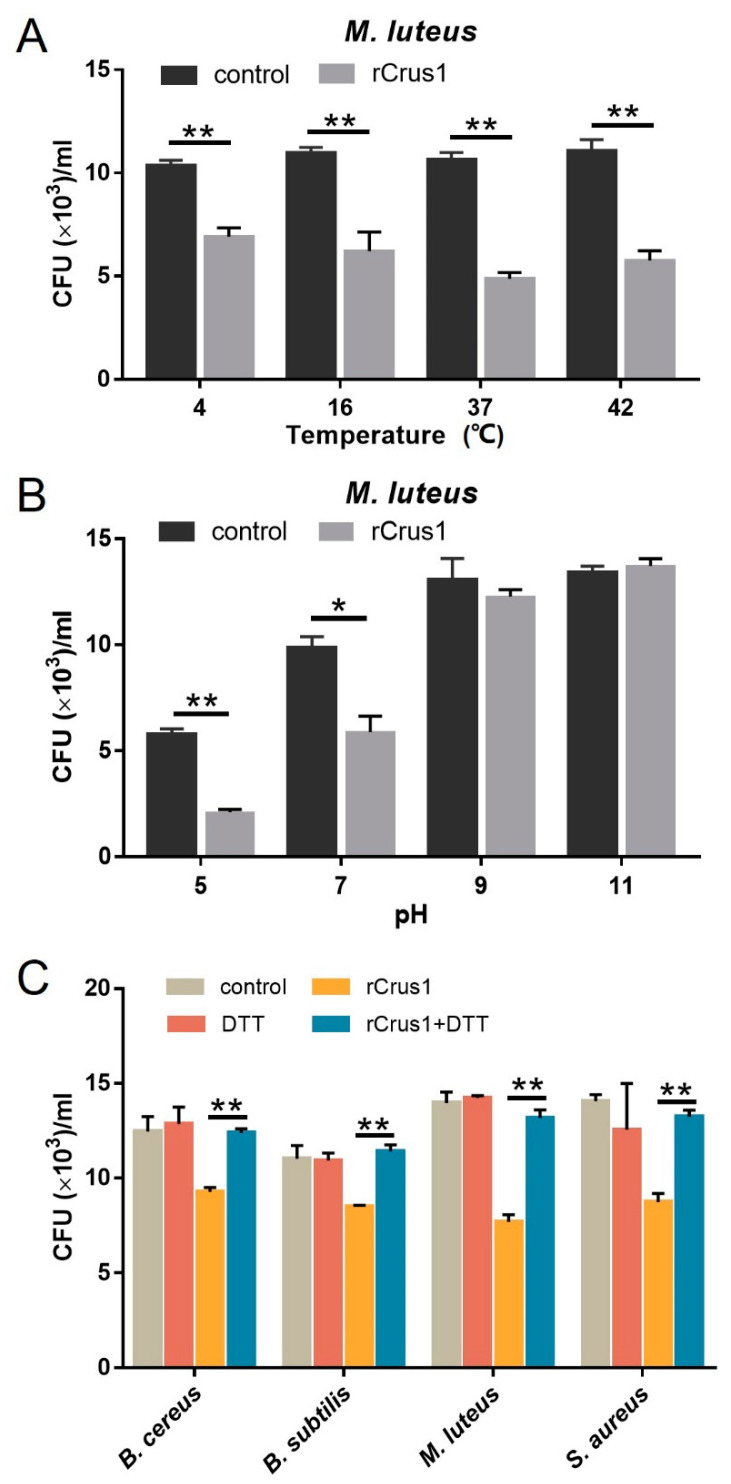
Effect of temperature, pH and disulfide bond on the antibacterial activity of rCrus1. (**A**) *Micrococcus luteus* was incubated with or without (control) rCrus1 (2.5 µM) at various temperatures for 2 h, and bacterial survival was determined by plate count. (**B**) *M. luteus* was incubated with or without (control) rCrus1 (2.5 µM) at various pH for 2 h, and bacterial survival was determined as above. (**C**) Bacteria were incubated with or without (control) rCrus1 (2.5 µM), DTT-treated rCrus1 (2.5 µM), or DTT for 2 h, and bacterial survival was determined as above. Values are shown as means ± SD (*N* = 3). N, the number of replicates. ** *p* < 0.01, * *p* < 0.05 (Student’s *t* test).

Recombinant Crus1 (rCrus1) was purified from *E. coli* as a His-tagged protein (Figure S1). The antibacterial activity of rCrus1 was tested against a variety of Gram-positive and Gram-negative bacteria, including those from deep sea environments, by measuring the minimal inhibitory concentration (MIC) and minimal bactericidal concentration (MBC) against each of the bacteria. As shown in Table 1, rCrus1 exhibited apparent inhibitory and killing activities against Gram-positive bacteria, but not against Gram-negative bacteria. The most potent activity was detected against *M. luteus*, with MIC and MBC values of 2.5 and 5 μM, respectively. Temperature dependence analysis showed that when rCrus1 was incubated with *M. luteus* at 4 °C, 16 °C, 37 °C, and 42 °C, the survival rates of the bacteria were similar (Figure 2A). pH dependence analysis showed that the bactericidal activity of rCrus1 against *M. luteus* was retained at pH 5 and 7, but completely lost at pH 9 and 11 (Figure 2B). In contrast, no apparent bactericidal activity of rCrus1 against the Gram-negative bacterium *Vibrio harveyi* was detected at either of these temperature/pH conditions (Figure S2). With *M. luteus* as the target bacterium, the killing effect of rCrus1 under the optimal condition (pH 7, 37 °C) was time-dependent (Figure S3). To examine whether the disulfide linkages were required for the bactericidal activity of rCrus1, the protein was treated with dithiothreitol (DTT), which reduces disulfide bond. The results showed that DTT treatment completely abolished the bactericidal effect of rCrus1 on *M. luteus* (Figure 2C).

### 2.3. Binding of rCrus1 to Bacterial Cell Wall Components and Its Effect on Bactericidal Activity

**Figure 3:**
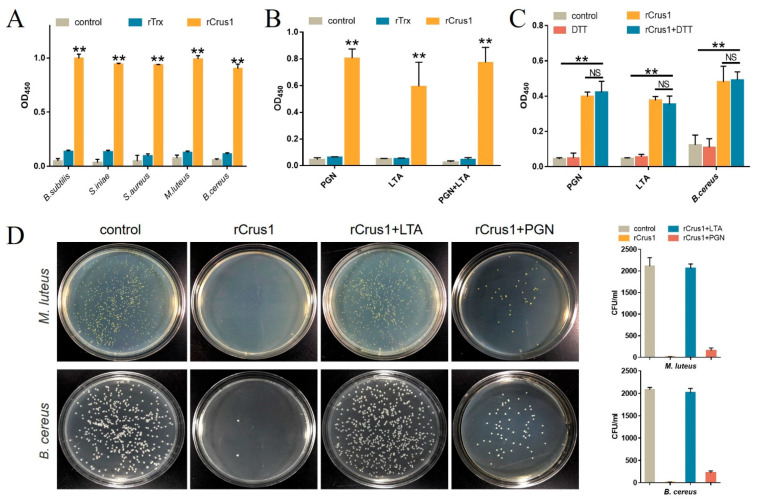
Binding of rCrus1 to bacteria and cell wall components. (**A**) Bacteria were incubated with rCrus1, recombinant Thioredoxin (rTrx), or phosphate buffer saline (PBS) (control), and the bound rCrus1 was detected by ELISA. (**B**) PGN and LTA were incubated with rCrus1, rTrx, or PBS, and the bound rCrus1 was detected as above. (**C**) PGN, LTA, and *Bacillus cereus* were incubated with or without (control) rCrus1, DTT-treated rCrus1, or DTT, and the bound rCrus1 was detected as above. Values are shown as means ± SD (*N* = 3). N, the number of replicates. ** *p* < 0.01, * *p* < 0.05, NS, not significant (Student’s *t* test). (**D**) *Micrococcus luteus* and *B. cereus* were treated with or without (control) rCrus1, rCrus1 plus PGN, or rCrus1 plus LTA for 1 h. The bacteria were plated on LB plates and observed after 20–24 h incubation. The number of colony-forming units (CFU) was determined and shown on the right panels.

Enzyme-linked immunosorbent assay (ELISA) showed that rCrus1 bound well to Gram-positive bacteria, including *M. luteus*, *S. aureus*, *B. subtilis*, *B. cereus* and *S. iniae* (Figure 3A). rCrus1, at the same concentration, also bound to Gram-negative bacteria, but the binding was much weaker than that to Gram-positive bacteria (Figure S4). Consistent with the relative strong binding between rCrus1 and Gram-positive bacteria, rCrus1 exhibited apparent and comparable bindings to peptidoglycan (PGN) and lipoteichoic acid (LTA) (Figure 3B). The binding of rCrus1 to the mixture of PGN and LTA was similar to that of the binding to PGN or LTA alone (Figure 3B). Treatment of rCrus1 with DTT had no significant effect on the binding of rCrus1 to PGN, LTA, or bacteria (Figure 3C). In the presence of exogenously added LTA or PGN, especially the former, the bactericidal effect of rCrus1 was markedly decreased (Figure 3D).

### 2.4. Effects of rCrus1 on the Morphology and Membrane Integrity of Bacteria

**Figure 4:**
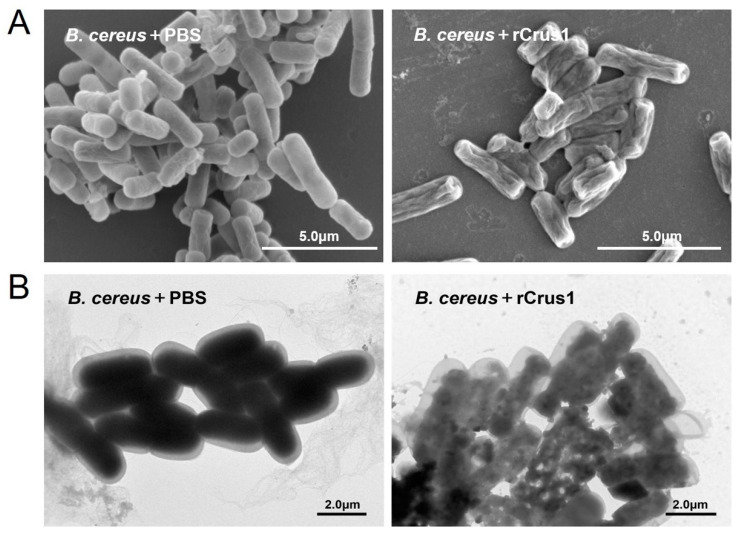
Morphological changes of the bacterial cells treated with rCrus1. *Bacillus cereus* was incubated with rCrus1 or PBS for 2 h, and the bacterial cells were inspected with a scanning (**A**) or transmission (**B**) electron microscope.

**Figure 5:**
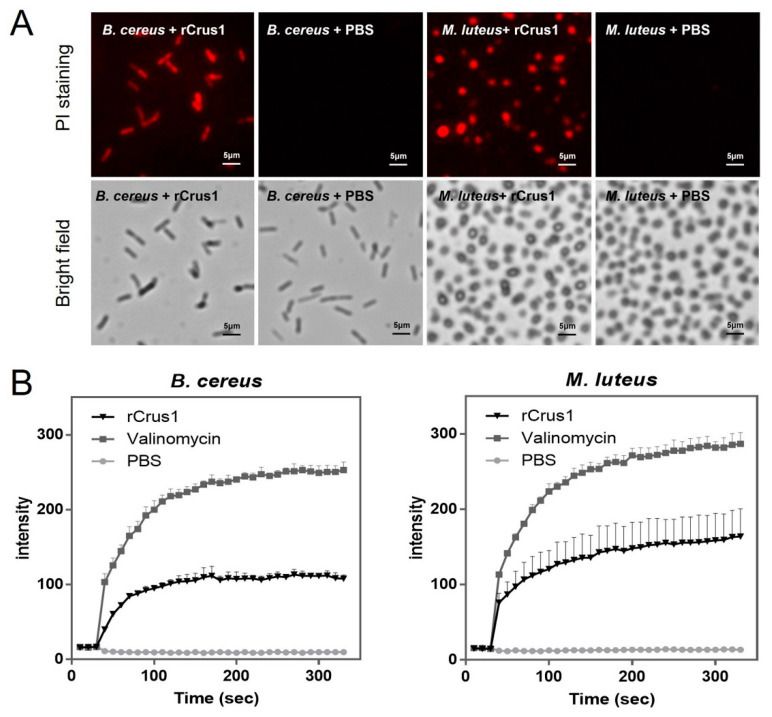
The effect of rCrus1 on bacterial cell membrane integrity. (**A**) *Bacillus cereus* and *Micrococcus luteus* were incubated with rCrus1 or PBS for 2 h. The cells were stained with PI and observed for PI uptake (upper panels) with a fluorescence microscope. The bright field image is shown in the lower panels. (**B**) *B. cereus* and *M. luteus* were pre-incubated with DiSC3(5) and then treated with rCrus1, valinomycin, or PBS, and the fluorescence of the cells was subsequently determined.

Electron microscopy showed that treatment of *B. cereus* with rCrus1 caused rapid changes in cell morphology. As revealed by scanning electron microscope (SEM) and transmission electron microscope (TEM), after 2 h treatment, the cells exhibited shrunken surface and much reduced cytoplasmic density (Figure 4A,B). Propidium iodide (PI) staining showed that after incubation of rCrus1 with *B. cereus* and *M. luteus*, a large amount of PI was able to penetrate into the bacteria cells (Figure 5A), suggesting damage of the cellular membrane. The ability of rCrus1 to mediate membrane damage was further investigated by using the membrane potential sensitive probe DiSC3 (5), which can monitor depolarization of the cell plasma membrane [28,29]. When DiSC3(5)-tr-eated bacteria were incubated with rCrus1, DiSC3(5) was found to be released from the cells, although the amount of released DiSC3(5) was much less than that released from the bacteria incubated with valinomycin, a strong depolarizer of membrane potential (Figure 5B). These results indicated that rCrus1 could cause membrane depolarization in a manner similar to, though in a lesser degree, valinomycin. In contrast, rCrus1 treatment of the protoplasts of *B. cereus* and *M. luteus* caused no apparent damage (Figure S5A). Consistently, rCrus1 did not bind to the protoplasts (Figure S5B).

### 2.5. The Conserved Cysteine Residues in the WAP Domain Are Essential to the Antimicrobial Activity of rCrus1

**Figure 6:**
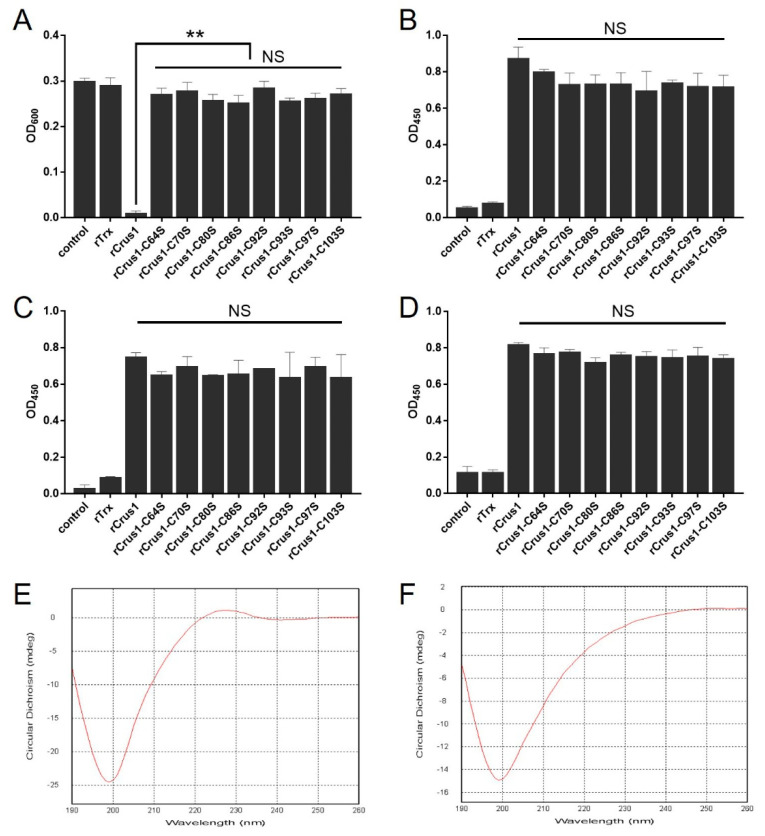
Microbial inhibitory and binding activity and structure characteristics of rCrus1 variants. (**A**) *Micrococcus luteus* was incubated with or without (control) 20 μM rCrus1, rCrus1 mutants, or rTrx for 18–20 h. Bacterial growth was then determined by measuring absorbance at OD_600_. (**B**–**D**) PGN (**B**), LTA (**C**), or *Bacillus cereus* (**D**) were incubated with or without (control) rCrus1, rCrus1 mutants, or rTrx, and the bound proteins were detected by ELISA. (E and F) Circular dichroism (CD) spectra of rCrus1 (**E**) and rCrus1-C103S (**F**) in PB buffer. Values are shown as means ± SD (*N* = 3). N, the number of replicate. ** *p* < 0.01, NS, not significant (one-way ANOVA).

To evaluate the functional importance of the conserved cysteine residues in the WAP domain, the eight Cys residues in this domain were mutated individually to Ser. The bactericidal activities of the resulting mutants, i.e., rCrus1-C64S, rCrus1-C70S, rCrus1-C80S, rCrus1-C86S, rCrus1-C92S, rCrus1-C93S, rCrus1-C97S, and rCrus1-C103S, were examined. None of the mutants exhibited apparent bactericidal activity at the MBC of rCrus1 (Table S1), or inhibited the growth of *M. luteus* even at the high concentration of 8 × MIC of rCrus1 (Figure 6A). However, all mutants were still able to bind to bacteria and bacterial cell wall components in a manner comparable to that of rCrus1 (Figure 6B–D). To examine whether the mutation changed the structure of rCrus1, the secondary structures of rCrus1 and rCrus1-C103S were subjected to circular dichroism (CD) analysis. Both rCrus1 and rCrus1-C103S showed a CD profile indicative of the formation of random coil structure; however, a fraction of the random coil differed slightly between rCrus1 and rCrus1-C103S (Figure 6E,F).

## 3. Discussion

Crustins are a large and diverse family of AMPs. In this study, we identified and analyzed a crustin, designated Crus1, from the shrimp of a deep-sea hydrothermal vent. Like typical crustins, Crus1 possesses a WAP domain, which contains eight conserved Cys capable of forming a four-disulfide core structure. It is interesting that, based on its cysteine-rich region [30], Crus1 was classified by phylogenetic analysis as a member of the Type I crustin, which has been mainly found in crabs and lobsters [31]. This observation of Crus1 is in contrast to that of the recently reported Re-crustin from the hydrothermal vent in MAR, which is a Type II crustin [27] and, as shown in our study, shares a low sequence identity with Crus1. These results suggest the possible existence of diverse forms of crustins in deep sea hydrothermal shrimp. Structural modeling showed that Crus1 formed mainly random coil, with very few α-helix and β-pleated sheet, which suggests a possibility that Crus1 may function via a unique mechanism.

The antimicrobial properties of crustins have been reported by many research groups [32,33]. Generally, crustins exhibit a broad-spectrum of antibacterial activities against Gram-positive and Gram-negative bacteria and fungi [34]. However, most members of the Type I crustins appear to have a spectrum of activity restricted to Gram-positive bacteria [19]. Similarly, in our study, we found that rCrus1 effectively killed Gram-positive bacteria from land, coastal waters, and deep sea, but had very limited killing effect on Gram-negative bacteria. The activity of rCrus1 was stable at acidic to neutral pH and over a wide range of temperatures, especially 4°C, which is close to the ambient temperature of the shrimp habitat. These results suggest that Crus1 likely functions as an active AMP under the native condition.

Binding to the target bacteria is a prerequisite for the antimicrobial activity of AMPs. In our study, rCrus1 bound strongly to Gram-positive bacteria as well as the major cell wall components of Gram-positive bacteria. We observed that the presence of added free PGN and LTA markedly reduced the bactericidal effect of rCrus1, suggesting that the binding between rCrus1 and the bacterial cell is likely mediated by PGN and LTA. Electron microscopy revealed that rCrus1-treated bacteria were shrunken and crinkled on the surface, resembling the formation of cracks on cells [35]. Consistently, PI staining indicated that rCrus1 induced membrane rupture in the bound bacterial cells, which was corroborated by the depolarization of membrane potential in rCrus1-treated bacteria. It is notable that, in contrast to the cell walled bacteria, the protoplasts of the bacteria were resistant to the binding and damage of rCrus1, which supported the above conclusion that it was the bacterial cell wall components, i.e., PGN and LTA, that rCrus1 interacted with directly. The importance of PGN and LTA is likely due to the reason that binding of AMPs to teichoic acids may initiate bacterial killing by facilitating the entry of the peptides toward the cytoplasmic membrane [36,37], and by building a poly anionic ladder, LTA and WTA may help poly cationic peptides, such as AMPs, to traverse from the outside to the cytoplasmic membrane [38]. The AMPs may further disrupt the cytoplasmic membrane by interfering with PGN biosynthesis [39]. It has been shown that the membrane bound PGN precursor lipid II could act as a docking moiety to attract cationic peptide to the bacterial membrane and promote peptide insertion into the membrane, leading eventually to membrane permeation [40,41].

For crustins, the WAP domain with its tetra-disulfide bond structure is thought to be vital to function [16,17]. In our study, we found that mutation of either of the eight cysteine residues in the WAP domain abolished the bactericidal activity of rCrus1, but neither of the mutations affected the ability of the protein to bind to bacteria or bacterial cell wall components. This finding indicates that bacterial binding and killing are via different mechanisms in rCrus1. Considering the importance of PGN and LTA in the binding of rCrus1 to bacteria, it is possible that rCrus1-bacteria interaction is mediated largely by ionic interaction, which is little affected by the Cys-to-Ser substitution, while bacterial killing is mediated by the interaction of the WAP domain with the bacterial membrane, which depends on the four-disulfide bonds. Circular dichroism showed that mutation of C103S caused a mild but distinct change in the secondary structure of rCrus1, which further supports the importance of the disulfide bonds of the WAP domain in the functioning of Crus1. It is possible that other residues besides these cysteines, such as those highly conserved in the WAP domain, may also paly vital roles in the structuring and functioning of Crus1.

In conclusion, our study demonstrates that, as an AMP, Crus1 binds bacteria probably via the bacterial cell wall components in a fashion that is independent of the WAP structure, but kills bacteria in a manner that requires the disulfide-based structural integrity of the WAP domain. These results add new insights into the immunological property and bactericidal mechanism of deep sea crustins.

## 4. Materials and Methods

### 4.1. Bacterial Strains and Culture Conditions

The bacteria used in this study are listed in Table 1. The Gram-positive bacteria (Bacillus subtilis WB800N, Bacillus subtilis G7, Bacillus wiedmannii SR52, Bacillus toyonensis P18, Staphylococcus aureus, Streptococcus iniae, and Micrococcus luteus) and the Gram-negative bacteria (*Escherichia coli*, *Edwardsiella tarda*, *Vibrio harveyi*, *Vibrio anguillarum*, and *Pseudomonas fluorescens*) have been reported previously [42,43,44,45]. Of these bacteria, *B. subtilis* G7, *B. wiedmannii* SR52, and *B. toyonensis* P18 are from deep sea hydrothermal vents. In addition, three other bacteria, i.e., *Pseudoalteromonas* sp., *Bacillus cereus* MB1, and *Bacillus* sp. are also from deep sea environments. *S. iniae* was cultured in TSB medium (Hopobio, Qingdao, China) at 28 °C. *E. tarda*, *B. subtilis* G7, *B. wiedmannii* SR52, *B. cereus* MB1, *Pseudoalteromonas* sp., and *Bacillus* sp. were cultured in marine 2216E medium (Hopobio, Qingdao, China) at 28 °C. All other bacterial strains were cultured in Luria-Bertani broth (LB) medium at 37 °C (for *E. coli*, *B. subtilis* WB800N, M. luteus and *S. aureus*) or 28 °C (for *P. fluorescens*, *V. anguillarum*, and *V. harveyi*). When used for determining the antibacterial activity of rCrus1, the bacteria were cultured in Mueller-Hinton broth (MHB) medium.

### 4.2. Bioinformatics Analysis and Structural Modeling of Crus1

The nucleotide sequence of Crus1 has been deposited to GenBank (accession number MW448473). The deduced amino acid sequence of Crus1 was analyzed with DNAMAN 6.0 (Lynnon Biosoft, San Ramon, CA, USA). Homology searches of deduced amino acid sequences were performed using the Protein BLAST algorithm of the NCBI. Signal peptide was identified using the SignalP program [46]. Multiple alignments of amino acid sequences were created with ClustalX 2.0 (SFI, Dublin, Ireland), and the output pattern was generated using DNAMAN 6.0 (Lynnon Biosoft, San Ramon, CA, USA). The neighbor-joining phylogenetic tree was constructed with MEGA 6.0 (Mega Limited, Auckland, New Zealand), and 1000 bootstraps were selected to assess reliability. The full-length atomic model of Crus1 was constructed with iterative template-based fragment assembly simulations using I-TASSER [47], and the spatial structure was edited with PyMOL 3.7 (Schrödinger, New York, NY, USA).

### 4.3. Protein Expression and Purification

To construct pETCrus1, the plasmid expressing rCrus1, the coding sequence of Crus1 without signal peptide and C-terminally tagged with six histidine residues was synthesized by BGI Technology (Beijing, China). The sequence was inserted into the expression plasmid pET28a (Sangon Biotech, Shanghai, China) at the NdeI/NotI sites, resulting in pETCrus1. pETCrus1 and pET32a (Novagen, Madison, WI, USA), which expresses rTrx, were separately introduced into *E. coli* BL21(DE3) (TransGen Biotech, Beijing, China) by transformation. The transformants were cultured in LB medium at 37 °C to OD600 0.6. Isopropyl-beta-d-thiogalactoside (IPTG) was added to the culture at a final concentration of 0.06 mM to induce protein expression. The culture was continued overnight at 16 °C with shaking (120 rpm), and the cells were then harvested by centrifugation. His-tagged rCrus1 and rTrx were purified as described previously [48]. Briefly, the cells were disrupted by sonication on ice, and the lysate was centrifuged to collect the supernatant. The His-tagged recombinant protein in the supernatant was purified under native conditions using nickel-nitrilotriacetic acid (Ni-NTA) columns (QIAGEN, Germantown, MD, USA) as recommended by the manufacturer. The protein was also treated with Triton X-114 to remove endotoxin as reported previously [49]. The purified protein was dialyzed against PBS for 36 h at 4 °C and concentrated with Amicon Ultra Centrifugal Filter (Millipore, Bedford, MA, USA). The purified protein was analyzed by sodium dodecyl sulfate-polyacrylamide gel electrophoresis (SDS-PAGE). The protein concentration was determined using the BCA Protein Assay Kit (Beyotime, Shanghai, China) according to the manufacturer’s instruction.

For the preparation of Crus1 mutants, site-directed serine substitutions of Cys64 (C64S), Cys70 (C70S), Cys80 (C80S), Cys86 (C86S), Cys92 (C92S), Cys93 (C93S), Cys97 (C97S), and Cys103 (C103S) were performed using the Q5 Site-Directed Mutagenesis Kit (New England Biolabs, Beverly, MA) according to the manufacturer’s instruction. The mutant proteins were expressed and purified as described above. The primers used in mutagenesis are shown in Table S2.

### 4.4. Antibacterial Activity Assay

The MIC and MBC were determined with the microdilution broth method [50]. In short, bacteria were cultured in MHB to an OD600 of 0.5–0.6 and diluted to 10^4^ CFU/mL. The bacteria were mixed with a 2-fold dilution series of rCrus1 in a 96-well microtiter plate. The plate was incubated at 37 °C for 18 h. MIC is defined as the lowest concentration of rCrus1 that rendered no visible bacterial growth. To determine the MBC, 10 μL of bacteria-rCrus1 mixture was removed from the above wells corresponding to 1 × MIC, 2 × MIC and 4 × MIC, and plated on MH agar plates. MBC is defined as the concentration of rCrus1 that killed 99.9% of bacteria after 18 h incubation. The assay was performed at least three times.

To examine the effect of temperature on rCrus1 activity, rCrus1 (1 × MIC) or PBS was incubated with 1 × 10^4^ CFU/mL *M. luteus* in MHB at 4 °C, 16 °C, 37 °C, or 42 °C for 2 h. Bacterial survival was then examined by plate count as described above. To examine the effect of pH on rCrus1 activity, rCrus1 or PBS was mixed with *M. luteus* as above and incubated in MHB (37 °C) at pH 5, 7, 9, or 11 for 2 h. Bacterial survival was determined as above. To examine the effect of disulfide bond elimination on rCrus1 activity, DTT (final concentration 50 mM) was added to rCrus1 in PBS and incubated for 30 min at 37 °C. The mixture was named DTT-treated rCrus1 and used immediately in the subsequent bactericidal assay. For the bactericidal assay, rCrus1 (2.5 µM), DTT-treated rCrus1 (2.5 µM rCrus1 plus 12.5 mM DTT), DTT (12.5 mM), or PBS (control) was incubated with *M. luteus* as above for 2 h. Bacterial survival was determined as above. To examine the effect of LTA and PGN on the bactericidal activity of rCrus1, LTA and PGN (Sigma, St Louis, MO, USA) (final concentration of 1 mg/mL) were each incubated with 1 × MBC rCrus1 for 1 h at room temperature, and then bacteria was added to the mixture. After incubation for 2 h, bacterial survival was determined as above by plate count. To examine the time-dependent bactericidal activity of rCrus1, *M. luteus* was cultured in MHB to an OD600 of 0.2 and diluted to 10^6^ CFU/mL in fresh MHB. rCrus1 (final concentration of 10 μM) was added to the bacterial dilution, followed by incubation at 37 °C for 5 h. Every 30 min, the number of bacteria was determined by plate count. All experiments were performed in triplicate.

### 4.5. Protein Binding to Bacteria and Cell Wall Components

Bacteria were cultured to an OD600 of 0.8 and resuspended in coating buffer (0.159% Na_2_CO_3_ and 0.293% NaHCO_3_, pH 9.6) to 10^8^ CFU/mL. LTA and PGN (Sigma, USA) were dissolved in coating buffer to 200 μg/mL. Binding of rCrus1 (20 μM) to bacteria and cell wall components was determined with ELISA as reported previously [51,52] with modifications. Briefly, a 96-well microtiter plate containing each of the bacteria or cell wall components (100 μL/well) was incubated overnight at 4 °C. The plate was washed 3 times with PBST (PBS with 0.05% Tween 20), and blocked with 200 μL 5% skim milk powder (Solarbio, Beijing, China) in PBST at 37 °C for 2 h. The plate was washed three times as above, and 10 μM protein (rCrus1 or rTrx) or PBS (control) was added to the wells. The plate was incubated at 37 °C for 1 h and washed as above. HRP-conjugated mouse anti-His antibody (1/1000 dilution) (ABclonal, Hubei, China) was added to the wells. The plate was incubated at 37 °C for 1 h and washed 5 times with PBST. Color development was performed using TMB substrate solution (Tiangen, Beijing, China) and terminated by adding ELISA stop solution (Solarbio, Beijing, China). The absorbance at 450 nm was measured using a multifunctional microplate reader. To examine the effect of disulfide bond elimination on the ability of rCrus1 to interact with bacteria and bacterial components, the binding assay was performed as above using rCrus1 (10 µM), DTT-treated rCrus1 (10 µM rCrus1 plus 50 mM DTT), DTT (50 mM), or PBS (control).

### 4.6. Electron Microscopy and PI Staining Assay

Electron microscopy was performed based on previous methods [53]. For microscopy with SEM (S-3400N, Hitachi, Tokyo, Japan), *B. cereus* MB1 and *M. luteus* were cultured in LB medium to logarithmic phase, and the cells were washed and resuspended in PBS to 1 × 10^6^ CFU/mL. The cells were pretreated with 1 × MBC rCrus1 or PBS at 37 °C for 2 h. After treatment, the cells were fixed with 5% glutaraldehyde in PBS (pH 7.4) for 2 h and dehydrated in a series of increased concentration of ethanol (30, 50, 70, 80, 90 and 100%) for 10 min at 4 °C. The cells were treated with isoamyl acetate for 10 min, critical point-dried (Hitachi-HCP, Hitachi, Japan), sputter-coated with platinum (MC1000, Hitachi, Japan) and examined with a SEM. For microscopy with TEM (HT7700, Hitachi, Tokyo, Japan), *B. cereus* and *M. luteus* were pretreated with rCrus1 or PBS as above. TEM microscopy was performed as previously reported [54]. For the PI assay, *B. cereus* MB1 and *M. luteus* were pretreated with rCrus1 or PBS as above. The sample was stained with a PI staining kit (BestBio, Shanghai, China) following the manufacturer’s instruction. The cells were observed with a fluorescence microscope (TiS/L100, Nikon, Tokyo, Japan).

### 4.7. Bacterial Cytoplasmic Membrane Depolarization

The cytoplasmic membrane depolarization activity of the rCrus1 was determined as reported previously d [29]. Briefly, *B. cereus* MB1 and *M. luteus* were grown at 37 °C to an OD600 of 0.6 and harvested by centrifugation. The cells were washed three times with HEPES buffer (5 mM HEPES with 20 mM glucose, pH 7.4), and resuspended in HEPES buffer containing 100 mM KCI to an OD600 of 0.05. DiSC3(5) (Macklin, Shanghai, China) was added to the bacterial suspension at a final concentration of 0.4 μM. The mixture was incubated in the dark for 30 min and then quenched at room temperature. rCrus1 (2 × MBC), valinomycin (a potassium ionophore), or PBS was added to the mixture. Membrane depolarization was monitored by observing change in the intensity of fluorescence (λex = 622 nm, λem = 670 nm).

### 4.8. Protoplast Preparation and Lysis Assay

Preparation of bacterial protoplast was performed as previously reported [55]. In short, *B. subtilis* G7 and *M. luteus* were cultured in LB broth to an OD600 of 0.9–1. The cells were pelleted by centrifugation at 12,000 rpm for 1 min. The cells were washed twice with pre-warmed (37 °C) steady buffer (20 mM sodium malate, 20 mM MgCl_2_, and 500 mM sucrose, pH 6.5). The cells were resuspended in steady buffer containing lysozyme (2.0 mg/mL) to an OD600 of 0.5–0.8 and incubated at 37 °C for 2–2.5 h. After washing twice with steady buffer, the cells were incubated with rCrus1 (1 × MBC) for 1 h at 37 °C. Triton X-100 (1%) was used as a positive control for maximal cell lysis. The OD600 of the protoplasts was measured and statistically calculated.

### 4.9. Immunofluorescence Microscopy

*B. subtilis* G7 and *B. subtilis* G7 protoplasts were diluted to 10^8^ CFU/mL with PBS and steady buffer, respectively. The cells were dropped onto adhesion microscope slides (CITOTEST, Jiangsu, China) at 4 °C and allowed to stand overnight. The slides were washed with PBS or steady buffer and incubated with rCrus1 (20 μM) at 37 °C for 1 h. The slides were washed as above, and anti-His-FITC antibody (Abcam, Cambridge, MA, USA) (1/200 dilution) was added to the slide. The slides were incubated at 37 °C for 2 h and washed as above. The slides were observed with a fluorescence microscope (Carl Zeiss LSM710, Jena, Germany).

### 4.10. Circular Dichroism (CD) Spectroscopy

CD spectroscopy was performed by Sangon Biotech Co., Ltd. (Shanghai, China). Briefly, the protein sample was diluted to 0.2 mg/mL with PB buffer (20 mM Na_2_HPO_4_·12H_2_O). The spectra were collected on a Chirascan Plus CD spectrometer (Applied Photophysics, Leatherhead, UK) using a 0.5 mm path cell. The data were obtained from 190 to 260 nm at an interval of 1.0 nm and a speed of 2 nm/s.

### 4.11. Statistical Analysis

Statistical analysis was carried out using GraphPad Prism 7.0 (GraphPad, San Diego, CA, USA). Statistical significance was determined with Student’s *t* test for two groups or one-way analysis of variance (ANOVA) for more than two groups. All data are presented as mean ± SD. *p* < 0.05 was considered statistically significant.

## Figures and Tables

**Table 1:** The minimal inhibitory concentration (MIC) and the minimal bactericidal concentration (MBC) of rCrus1 against Gram-positive and Gram-negative bacteria.

Bacteria	MIC (μM)	MBC (μM)
**Gram-positive**		
*Bacillus subtilis* WB800N	20	40
*Bacillus subtilis* G7	40	40
*Bacillus wiedmannii* SR52	20	40
*Bacillus cereus* MB1	20	40
*Bacillus toyonensis* P18	30	60
*Bacillus sp*	30	60
*Micrococcus luteus*	2.5	5
*Staphylococcus aureus*	10	20
*Streptococcus iniae*	15	30
**Gram-negative**		
*Escherichia coli*	—	—
*Vibrio harveyi*	—	—
*Edwardsiella tarda*	—	—
*Vibrio anguillarum*	—	—
*Pseudoalteromonas sp*	≥200	—
*Pseudomonas fluorescens*	—	—

—: No inhibitory or bactericidal activity was detected at the tested concentrations (1.25–60 μM).

## Data Availability

The Crus 1 sequence data of this study are available from GenBank under the accession number MW448473.

## Supplementary Materials

The following are available online at https://www.mdpi.com/1660-3397/19/3/176/s1, Figure S1: SDS-PAGE analysis of rCrus1, Figure S2. Effect of temperature and pH on the antibacterial activity of rCrus1 against *Vibrio harveyi*, Figure S3. Time-dependent bactericidal activity of rCrus1 against *Micrococcus luteus*, Figure S4. Binding of rCrus1 to Gram-negative bacteria, Figure S5: The potential effect of rCrus1 on bacterial protoplasts, Table S1. Bactericidal activity of rCrus1 variants, Table S2. Primers used in point mutation.
