# Supplementary material for: A Crustin from Hydrothermal Vent Shrimp: Antimicrobial Activity and Mechanism
Source: Mar Drugs. 2021 Mar 23;19(3):176. doi: 10.3390/md19030176 (PMC8005205; doi:10.3390/md19030176)

Supplementary data

Table S1. The bactericidal activity of rCrus1 variants.

**Crustin**	***B. cereus***	***M. luteus***
control	－	－
rTrx	－	－
rCrus1	＋	＋
rCrus1-C64S	－	－
rCrus1-C70S	－	－
rCrus1-C80S	－	－
rCrus1-C86S	－	－
rCrus1-C92S	－	－
rCrus1-C93S	－	－
rCrus1-C97S	－	－
rCrus1-C103S	－	－

＋: Bactericidal activity was detected at 1×MBC of rCrus1.

－: No bactericidal activity was detected at 1×MBC of rCrus1.

Table S2. Primers used in point mutation.

Primer name	Sequence (5'-3')
Cys64-F	5'-GGAACACAAAGGAGAGTGTCCTGAGGTGCG-3'
Cys64-R	5'-CGCACCTCAGGACACTCTCCTTTGTGTTCC-3'
Cys70-F	5'-GTCCTGAGGTGCGACCCAGCCCAGGGATAAAGTTC-3'
Cys70-R	5'-GAACTTTATCCCTGGGCTGGGTCGCACCTCAGGAC-3'
Cys80-F	5'-GATAAAGTTCTCCCCCCAACTGAGTCCCCATGAC-3'
Cys80-R	5'-GTCATGGGGACTCAGTTGGGGGGAGAACTTTATC-3'
Cys86-F	5'-GTCCCCATGACGGTCACAGCAAACGCAACG-3'
Cys86-R	5'-CGTTGCGTTTGCTGTGACCGTCATGGGGAC-3'
Cys92-F	5'-GCAAACGCAACGAAAAGAGTTGCTACGACTCTTGCC-3'
Cys92-R	5'-GGCAAGAGTCGTAGCAACTCTTTTCGTTGCGTTTGC-3'
Cys93-F	5'-CAAACGCAACGAAAAGTGTAGCTACGACTCTTGCCTCG-3'
Cys93-R	5'-CGAGGCAAGAGTCGTAGCTACACTTTTCGTTGCGTTTG-3'
Cys97-F	5'-GTTGCTACGACTCTAGCCTCGAGCACCACG-3'
Cys97-R	5'-CGTGGTGCTCGAGGCTAGAGTCGTAGCAAC-3'
Cys103-F	5'-AGCACCACGCCAGCAAGCTCGCCTCCAATGCACAT-3'
Cys103-R	5'-ATGTGCATTGGAGGCGAGCTTGCTGGCGTGGTGCT-3'

Figure S1. SDS-PAGE analysis of rCrus1. Purified rCrus1 (lane1) was analyzed by SDS-PAGE and viewed after staining with Coomassie brilliant blue R-250.

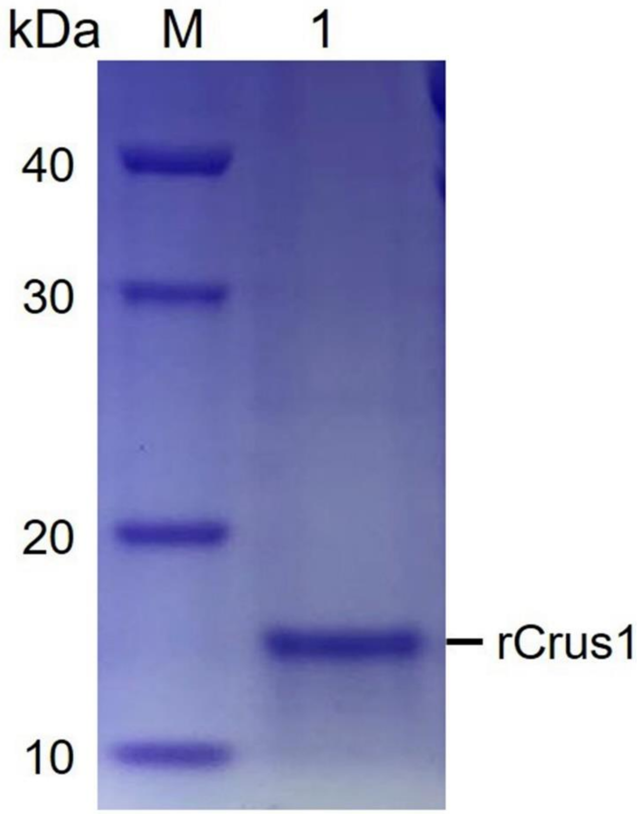

Figure S2. Effect of temperature (A) and pH (B) on the antibacterial activity of rCrus1 against *Vibrio harveyi*. (A) *V. harveyi* was incubated with or without (control) rCrus1 at various temperatures for 2 h, and bacterial survival was determined by plate count. (B) *V. harveyi* was incubated with or without (control) rCrus1 at various pH for 2 h, and bacterial survival was determined as above. Values are shown as means ± SD (N = 3). N, the number of replicate.

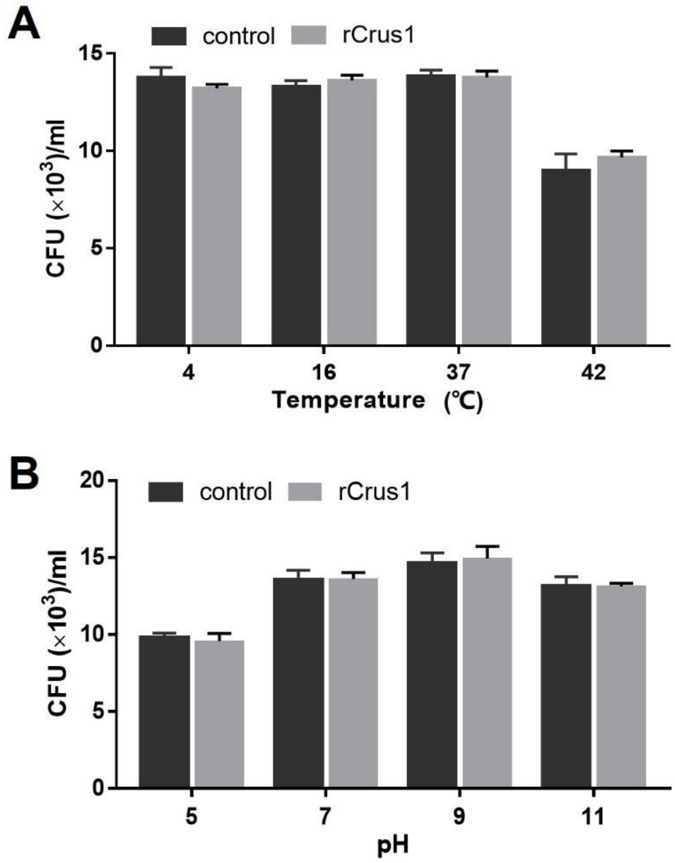

Figure S3. Time-dependent bactericidal activity of rCrus1 against *Micrococcus luteus*. *M. luteus* was grown in MHB supplemented with or without (control) rCrus1 at the condition of pH 7 and 37°C. Bacterial survival was determined at various time points by plate count. Values are shown as means ± SD (N = 3). N, the number of replicate.

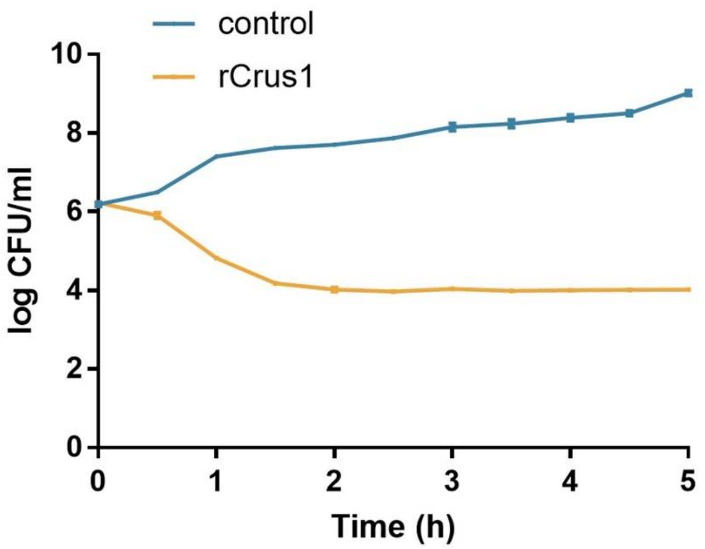

Figure S4. Binding of rCrus1 to Gram-negative bacteria. *Escherichia coli*, *Edwardsiella tarda*, *Pseudomonas fluorescens*, *Vibrio anguillarum*, and *Vibrio harveyi* were incubated with rCrus1, rTrx, or PBS (control) for 1 h, and the bound rCrus1 was detected by ELISA. Values are shown as means ± SD (N = 3). N, the number of replicate. $^{**}P < 0.01$, (Student's t test).

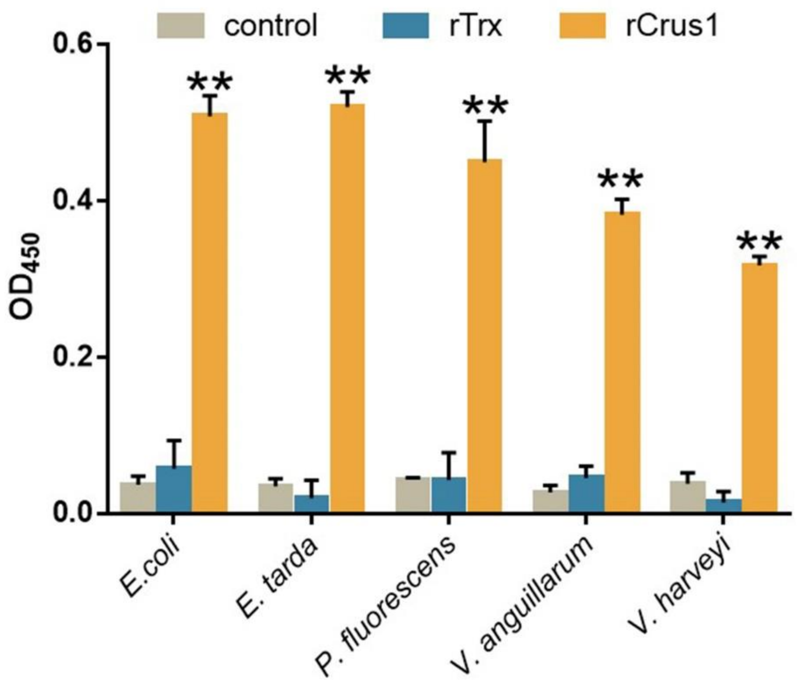

Figure S5. The potential effect of rCrus1 on bacterial protoplasts. (A) The protoplasts of *Bacillus subtilis* and *Micrococcus luteus* were incubated with rCrus1, Trion X-100 (positive control), or PBS for 1 h and then measured for absorbance at OD_{600}. Values are shown as means ± SD (N = 3). N, the number of replicate. $^{**}P < 0.01$, $^{*}P < 0.05$ (Student's t test). (B) *B. subtilis* (Ba) and *B. subtilis* protoplasts (Bb) were pretreated with His-tagged rCrus1 or rTrx (control) for 1 h, and the bound protein was detected with anti-His-FITC antibody and observed with a fluorescence microscope.

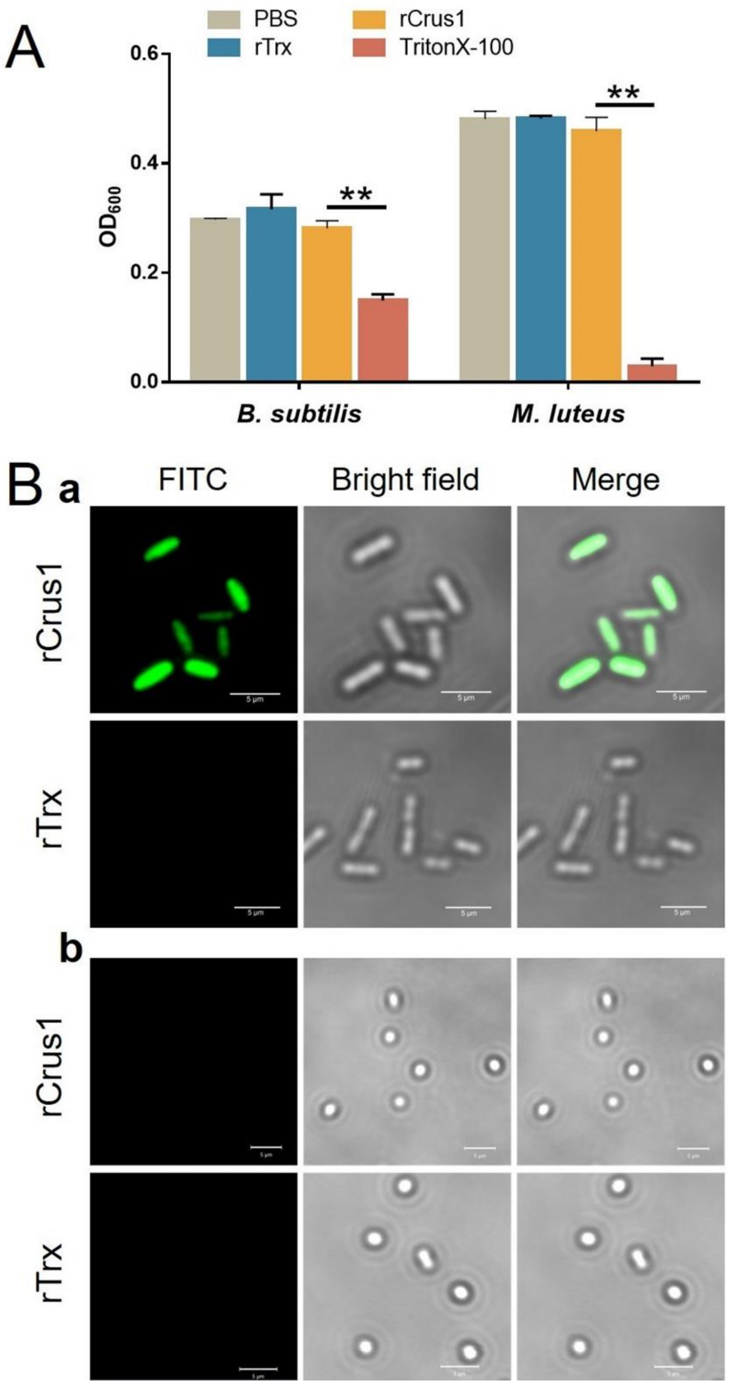

Supplement: Supplementary file 1 [file marinedrugs-19-00176-s001.pdf]
